# Melt Blending Modification of Commercial Polystyrene with Its Half Critical Molecular Weight, High Ion Content Ionomer, Poly(styrene–*ran*–cinnamic Acid) Zn Salt, toward Heat Resistance Improvement

**DOI:** 10.3390/polym12030584

**Published:** 2020-03-05

**Authors:** Zixin Yu, Jie Wang, Peihua Li, Dachuan Ding, Xuan Zheng, Chuanqun Hu, Zhinan Gao, Tao Hu, Xinghou Gong, Chonggang Wu

**Affiliations:** Hubei Provincial Key Laboratory of Green Materials for Light Industry, Collaborative Innovation Center of Green Light-weight Materials and Processing, and School of Materials and Chemical Engineering, Hubei University of Technology, Wuhan 430068, China; zixin_yu_1008@163.com (Z.Y.); jack_w929@163.com (J.W.); lphmeme@163.com (P.L.); ddcmck@outlook.com (D.D.); hanksmithmikeyou@163.com (X.Z.); nanohu@126.com (C.H.); zhinan_gao@163.com (Z.G.); hutao@mail.hbut.edu.cn (T.H.); gongxh@hbut.edu.cn (X.G.)

**Keywords:** polystyrene, heat resistance, blending modification, poly(styrene–*ran*–cinnamic acid), ionomer

## Abstract

A half-critical weight-average molecular weight (${\bar{M}}_{w}$) (approximately 21,000 g mol^−1^), high-ion-content Zn-salt poly(styrene–*ran*–cinnamic-acid) (SCA–Zn) ionomer was successfully synthesized by styrene–cinnamic-acid (10.8 mol %) copolymerization followed by excess-ZnO melt neutralization. At 220 °C, the SCA–Zn’s viscosity was only approximately 1.5 magnitude orders higher than that of commercial polystyrene (PS) at 10^2^ s^−1^, and the PS/SCA–Zn (5–40 wt %) melt blends showed apparently fine, two-phased morphologies with blurred interfaces, of which the 95/5 and 90/10 demonstrated Han plots suggesting their near miscibility. These indicate that any PS–(SCA–Zn) processability mismatch was minimized by the SCA–Zn’s half-critical ${\bar{M}}_{w}$ despite its dense ionic cross-links. Meanwhile, the SCA–Zn’s Vicat softening temperature (*VST*) was maximized by its cross-linking toward 153.1 °C, from that (97.7 °C) of PS, based on its half-critical ${\bar{M}}_{w}$ at which the ultimate glass-transition temperature was approximated. Below approximately 110 °C, the PS/SCA–Zn (0–20 wt %) were seemingly miscible when their *VST* increased linearly yet slightly with the SCA–Zn fraction due to the dissolution of the SCA–Zn’s cross-links. Nevertheless, the 60/40 blend’s *VST* significantly diverged positively from the linearity until 111.1 °C, revealing its phase-separated morphology that effectively enhanced the heat resistance by the highly cross-linked SCA–Zn. This work proposes a methodology of improving PS heat resistance by melt blending with its half-critical ${\bar{M}}_{w}$, high-ion-content ionomer.

## 1. Introduction

Owing to its relatively high strength and modulus, superior electrical insulation, satisfactory dyeability, and excellent processability, polystyrene (PS) has widely been used in construction, automotive, packaging, etc. industries. However, the presence of bulky, rigid pendant phenyl groups as well as weak, nonpolar chain-segmental interactions in PS dictates its poor impact toughness, which, along with its not high enough heat resistance (Vicat softening temperature, *VST* and glass transition temperature, *T*_g_ of both approximately 100 °C), limits its extension to engineering applications. Numerous modifications are conducted to improve the heat resistance of PS, e.g., blending, copolymerization, grafting, filling, interpenetration, their combinations, etc. When PS is melt-blended with more rigid resins such as poly(bisphenol-A carbonate) (PC) [1], brominated PS [2], etc., the *VST* of the blends is enhanced effectively; the blending of PS with more flexible polymers such as elastomers [3,4], high-density polyethylene [5], poly(lactic acid) [6], etc. may increase the blends’ impact toughness, while their *VST* is reduced. Copolymerizing PS with small amounts (5–25 mol %) of stiffer cinnamic acid (CA) [7], α-methylstyrene [8], *N*-phenylmaleimide [9], 4-vinylbenzocyclobutene [10], cinnamonitrile [11], maleimide [12], maleic anhydride [12,13], 3-sulfopropyl methacrylate Na salt [14], etc. significantly raises the *T*_g_ or *VST* of the copolymers, despite a possible degree of deterioration in their mechanical property(ies). Upon solution grafting with methyl methacrylate [15] and 1,4-dimethyl-2,5-dichloromethyl benzene [16] polar and/or rigid groups, PS exhibits an enhanced *T*_g_. Melt-filling PS with different fractions (3–15 wt %) of Fe powder [17], nano-TiO_2_ [18], nano-Ca_3_(PO_4_)_2_ [19], and nano-pseudoboehmite [20] increases its *VST* little with a possible decrease in its tensile strength or impact toughness, which is due presumably to weak PS–inorganic interfacial interactions; likewise, if it solution-fills with montmorillonite, PS simply displays a slightly raised *VST* [21], whereas filling PS, in situ during polymerization, with organophilic montmorillonite [22], -graphite [23], and -graphene nanosheets [24] considerably heightens the heat resistance of the composites as a result of their improved interfacial adhesion. Initiating the polymerization of styrene in a polyurethane network obviously strengthens the heat resistance of the PS as well by producing interpenetrating polymer networks [25]. Of course, combined modifications, e.g., an Al_2_O_3_-filling and PC-blending coupling of PS [26], may also boost its heat resistance distinctly.

Regarding PS blending modification, although researchers investigated the effects of sulfonated polystyrene Na salt (SPS–Na) content and SPS–Li ion content on the mechanical properties and morphology, respectively, of PS/SPS–Na [27] and PS/SPS–Li [28] solution blends, no methodology of blending of PS with its ionomer has been probed toward the effective enhancement of its heat resistance under a practical melt blending regime. For this purpose, the PS ionomer has to be of dense ionic cross-linking to give a much higher *T*_g_ than the PS, while of the lowest possible molecular weight to minimize a processability mismatch with the PS but not to essentially compromise (i.e., reduce) the *T*_g_. To address the issue, a low (i.e., half critical) molecular weight, high ion content poly(styrene–*ran*–cinnamic acid) (SCA) Zn salt, SCA–Zn, is successfully synthesized in this work and then melt-blended with commercial PS at increasing mass ratios. The effect of SCA–Zn content on the heat resistance, against those on the mechanical and rheological properties, is subsequently studied of the PS/SCA–Zn blends.

## 2. Materials and Methods

### 2.1. Materials

Styrene (≥98.0%), NaOH (≥96.0%), ethanol, anhydrous (≥99.7%), tetrahydrofuran (THF) (≥99.0%), xylene (≥99.0%), all of analytical reagent (AR), potassium biphthalate (working chemical, ≥99.9%), and phenolphthalein (indicator grade) were purchased from Sinopharm (Shanghai) Chemical Reagents Co., Ltd., China. Na_2_SO_4_, anhydrous (AR, ≥99.0%) was supplied by Tianjin City Fuchen Chemical Reagents Plant, China. A poly(vinyl alcohol) (PVA) (20–88(L), ≥93.5%) was obtained from Anhui Province Wanwei New, High-property Materials Co., Ltd., China. (Di)benzoyl peroxide (BPO) (AR, ≥98.0%) was acquired from Shanghai Hengli Fine Chemicals Co., Ltd., China. (Trans-)CA (AR, 99.0%) was provided by Shanghai Macklin Biochemical Co., Ltd., China. THF (standard for GC, >99.9%) and KBr (spectral reagent, 99.0%) were offered by Aladdin Industrial Corp., China. Chloroform-*d* and ZnO, both of AR and ≥99.0%, were afforded by Shanghai Aladdin Bio-Chem Technology Co., Ltd., China. KOH (AR, ≥85.0%) was received from Tianjin City Yongtai Chemical Reagents Co., Ltd., China. A commercial PS resin (injection grade, GPPS 666D) was purchased from Sinopec Yanshan Petrochemical Co. Distilled water was homemade in our laboratory using a stainless-steel water distiller.

Prior to its use, styrene was purified by extractions with a 5 wt % of NaOH aqueous solution to remove any polymerization inhibitor(s) and impurities and then with distilled water to eliminate the residual NaOH, followed by drying with anhydrous Na_2_SO_4_. All of the other chemicals were used as received without any further purification unless otherwise specified below.

### 2.2. Suspension Free-Radical Copolymerization of Styrene and CA

Styrene was copolymerized with 20 wt % of CA using a suspension method. An overhead stirrer as well as a laboratory condenser was fitted to a 1 L three-necked round-bottom flask clamped and immersed in a room-temperature (RT) oil bath; then, 400 mL of distilled water and 0.72 g of the PVA dispersant were placed into the flask. After the PVA was swollen in water for 15 min, the quiescent mixture was stirred at 250 rpm while the bath gradually heated until the PVA was well dissolved to form a clear solution. The (bath) temperature was further increased to and stabilized at 120 °C to ensure a steady reflux of the solution, when 99 mL of (purified) styrene monomer and approximately 3.3750 g of BPO initiator were added to the flask. Subsequently, 22.5 g of CA comonomer was immediately added into the stirred suspension, and the copolymerization reaction was conducted for 5 h. At this juncture, if small solid particles were present and felt hard in a small amount of reaction suspension sampled with a dropper into some water, the system was therefore allowed to react for another 1 h. The resulting suspension, upon cooling to RT, was Büchner filtrated to obtain a white pellet product. The product was transferred to a 1 L beaker, soaked there in 200–300 mL of stirred distilled water for 5 min, and then Büchner filtered off; such a process was repeated at least three times. Afterwards, a similar washing process was repeated at least three times with stirred anhydrous ethanol as the soaking solvent. Finally, the fully washed product was air-dried in a fume hood for at least 3 days and subsequently vacuum-dried at 75 °C for at least 24 h to obtain a pellet resin, supposedly SCA.

### 2.3. Gel Permeation Chromatography (GPC)

To ascertain if it had an as low as half critical ${\bar{M}}_{w}$, the (potential SCA) product resin as synthesized above was measured at 25 °C using a gel permeation chromatograph (Agilent, PL-GPC 50, Palo Alto, CA, USA). THF was used as the eluent at a constant flow rate of 1.0 mL min^−1^ and PS resins of monodisperse molecular weights (up to 250,000 g mol^−1^) as the standards. For comparison, the as-purchased commercial PS (GPPS 666D) was analyzed similarly, but with a different chromatograph (Waters, Waters1525, Milford, MA, USA) and using PS-resin standards of up to 1,200,000 g mol^−1^ molecular weight.

### 2.4. Acid–Base Titration

To determine the possible carboxylic-acid group (i.e., CA) content of the product resin, a xylene solution (approximately 8.0 × 10^−3^ g mL^−1^) of an accurately weighed mass of its well-dried sample was titrated in an Erlenmeyer flask against a KOH ethanol solution from a basic buret. The KOH solution, with a nominal concentration of 1.0 × 10^−2^ M, was in turn titrated in situ to evaluate its effective concentration with a precise amount of potassium-biphthalate working chemical of approximately 1.0 × 10^−3^ g mL^−1^ in ethanol in another Erlenmeyer flask. For both the titrations, a phenolphthalein indicator solution in an ethanol/distilled water (19/1 *v/v*) mixed solvent (approximately 1.0 × 10^−2^ g mL^−1^) was used to identify the first occurrence of a reddish color as the equivalence point. Two samples of the product resin were subjected to the titration: one was the as-synthesized, in which there might be both copolymerized and free, unreacted CA species, and the other was purified by reprecipitation from xylene into a large amount of ethanol followed by filtration, washing, and vacuum drying at 75 °C for at least 24 h, where there should primarily be copolymerized CA. The CA contents (mol %), *c*_as_ and *c*_CA_, of the as-synthesized and purified resin samples, respectively were therefore calculated using the same equation,

(1) $$c_{\mathrm{as}}\;\mathrm{or}\;c_{\mathrm{CA}}=\frac{cV}{cV+\frac{m-M_{\mathrm{CA}}cV}{M_{\mathrm{St}}}}\times 100\;\mathrm{mol}\%$$

where *c* is the effective molar concentration (mol L^−1^ or M) of the KOH ethanol solution, *V* is the volume (L) of the KOH solution consumed until the equivalence point of the sample xylene solution titration, *m* is the accurately weighed mass (g) of the well-dried sample, and M_St_ and M_CA_ are the molar masses (g mol^−1^) (i.e., 104.15 and 148.16), respectively, of styrene and CA. To minimize any uncertainty of the results, the arithmetically mean *c*_as_ and *c*_CA_ values from three parallel titrations, respectively, were taken as the *c*_as_ and *c*_CA_ of the resin synthesized for analysis.

### 2.5. Melt Neutralization of the Potential S with Excess ZnO

If *c*_as_ > *c*_CA_ ≫ 0 mol % in Section 2.4, then the product resin preliminarily was assumed to be SCA. Practical melt neutralization was hence performed of the (potential) as-synthesized SCA with ZnO. According to a theoretical 2/1 (mol/mol) reaction of the CA units and ZnO, the stoichiometric amount (g) of ZnO, $m_{\mathrm{ZnO}}^{0}$, required for the neutralization was evaluated by the following equation,

(2) $$m_{\mathrm{ZnO}}^{0}=\frac{{mc}_{\mathrm{as}}M_{\mathrm{ZnO}}}{{2[M}_{\mathrm{St}}{+(M}_{\mathrm{CA}}-M_{\mathrm{St}}{)c}_{\mathrm{as}}]}$$

where *m* and *c*_as_ are the mass (g) and CA content (mol %) of the as-synthesized SCA, respectively, and M_ZnO_, M_St_, and M_CA_ are the molar masses (g mol^−1^) (i.e., 81.41, 104.15, and 148.16), respectively, of ZnO, styrene, and CA. To enhance the possible degree of neutralization, the actual feed (g) of ZnO, $m_{\mathrm{ZnO}}$, was made excessive by

(3) $$m_{\mathrm{ZnO}}=2.5m_{\mathrm{ZnO}}^{0}$$

Therefore, the as-synthesized SCA and ZnO, at a feed ratio of *m*/*m*_ZnO_ (cf. Equations (2) and (3)), were melt-reacted at 190 °C in a plasticorder (Harbin Harp Electrical Technology Co., Ltd., China, RM-200C) for 15 min at a rotor speed of 80 rpm to produce presumably an ionomer, SCA–Zn, which was then ground into a powder with a laboratory grinder (Yongkang City Boou Hardware Factory, China, 400Y).

### 2.6. Fourier Transform Infrared (FTIR) Spectroscopy

To reveal success in synthesizing SCA and further preparing SCA–Zn, the FTIR absorption spectrum of the (potential) purified SCA, as well as of the (potential) SCA–Zn similarly purified to the SCA, was compared in a mid-IR range of 2000–1200 cm^−1^ with that of the PS (GPPS 666D) purified likewise. An FTIR spectrometer (Bruker, Tensor II, Billerica, MA, USA) was employed, in the transmission mode, to collect at RT under a N_2_ atmosphere the spectra of a 4 cm^−1^ resolution based on 24 scans. The samples were prepared as KBr pellets by grinding together, using a pestle and mortar, approximately 2.0 mg of the well-dried resin powders and approximately 50 mg of KBr dried with an infrared baking lamp followed by RT compression of the mixture powders on a hydraulic press.

### 2.7. ^1^H Nuclear Magnetic Resonance (NMR) Spectroscopy

To verify that the SCA and SCA–Zn syntheses were successful, an NMR spectrometer (Bruker, Avance III HD 400 MHz, Billerica, MA, USA) was used to collect the ^1^H solution-state NMR spectra of the (potential) purified SCA and -SCA–Zn against the purified PS at 400 MHz with respect to a tetramethylsilane internal standard. Small amounts (5–10 mg) of the well-dried samples were fully dissolved in 0.5 mL of chloroform-*d* in an NMR tube to form a homogeneous solution of 10–20 mg mL^−1^.

### 2.8. Thermogravimetric Analysis (TGA)

As additional evidence of the successful syntheses, the thermal decomposition behaviors of the (potential) purified SCA and SCA–Zn were studied relative to the purified PS using a thermogravimetric–differential thermal simultaneous analyzer (TA Instruments, SDT Q600, Milford, MA, USA). Small amounts (approximately 3.0 mg) of the well-dried samples were placed in a (disposable, Al_2_O_3_) crucible on one tray of the thermal balance, which, along with an empty crucible on the other tray, was then heated from 30 to 600 °C at a rate of 20 °C min^−1^ under a N_2_ atmosphere of 100 mL min^−1^. The temperatures at which a 5% weight loss of the samples occurred were defined as their thermal decomposition temperatures (*T*_d_’s).

### 2.9. Intrinsic Viscosity Measurement

To further corroborate the success in the syntheses, an Ubbelohde viscometer was used to measure the intrinsic viscosity of the (likely) purified SCA–Zn as opposed to the (likely) purified SCA following the ISO 1682-1 testing standard. For the runs, either of the well-dried samples was dissolved at 25 ± 0.1 °C in a THF/distilled water (9/1 *v/v*) mixed solvent to prepare solutions of 5 decreasing concentrations, i.e., 10/10, 7/10, 6/10, 5/10, and 4/10 of 0.01 g mL^−1^.

### 2.10. Melt Blending of PS and the Zn-Salt SCA Ionomer

The as-purchased PS and the as-prepared SCA–Zn, both vacuum-dried at 75 °C for 12 h if necessary, were melt-blended in the plasticorder at 5 decreasing mass ratios of 100/0 (i.e., plasticated PS), 95/5, 90/10, 80/20, and 60/40 at 220 °C for 15 min at a rotor speed of 80 rpm. Then, the PS/SCA–Zn blends (including the plasticated PS) obtained were ground into powders with the laboratory grinder.

### 2.11. Oscillatory Shear Rheometry

A rotational rheometer (TA Instruments, DHR-2, Milford, MA, USA), with a 25-mm diameter, 1.0-mm separation parallel-plate fixture, was employed in the oscillatory shear mode to conduct frequency (10^−1^–10^2^ rad s^−1^) sweep experiments of the SCA, SCA–Zn, and PS/SCA–Zn blends (including the plasticated PS) at 220 °C under a N_2_ atmosphere. A small strain-amplitude of 0.5% was applied to ensure the linear viscoelasticity of the materials investigated. To minimize the effects of the sample preparation history, the as-synthesized pellet SCA used was plasticated and ground into a powder in light of the SCA–Zn neutralization conditions, and the SCA–Zn was used as prepared. Then, amounts (approximately 1.5 g) of all of the well-dried sample powders were compression-molded at 10 MPa, at 260 °C for the SCA–Zn and 220 °C for the others, into a discoid specimen approximately 25 mm in diameter and 2.0 mm in thickness.

### 2.12. Optical Microscopy

A single particle (approximately 3.0 mg) of each of the well-dried PS/SCA–Zn blends (including plasticated PS) powders was sandwiched between a glass slide and a cover glass. The sandwich was placed into a heating stage (Linkam, THMS 600, Tadworth, UK) maintained at 220 °C by a temperature controller, whose central sample layer was then stepwise melt-pressed into a near clear film of approximately 20–50 µm in thickness, until the stage temperature rebounded to and equilibrated at 220 °C. The phase morphology of the sample was subsequently observed on an optical microscope (Leica, DM 2500P, Wetzlar, Germany) under an objective magnification of 20, with its image taken using a digital camera connected to the microscope and interfaced with a microcomputer.

### 2.13. Differential Scanning Calorimetry (DSC)

The *T*_g_ values of the plasticated SCA, as-prepared SCA–Zn, and PS/SCA–Zn blends (including the plasticated PS) were measured according to the ISO 11357 testing standard using a differential scanning calorimeter (Perkin Elmer, DSC 8000, Waltham, MA, USA) under a N_2_ atmosphere of 20 mL min^−1^, upon its calibrations in both temperature and heat flow with an indium standard. Small amounts (approximately 5.0 mg) of the well-dried sample powders, encapsulated into a flat-bottomed aluminum pan, were enclosed in the DSC sample cell while an empty pan–lid capsule was maintained in the reference cell. Then, both the cells were heated from 30 to 200 °C to minimize the effects of the sample preparation history, followed by cooling to 30 °C and finally heating again to 200 °C, all at a rate of 20 °C min^−1^. Prior to the sample runs, a baseline was acquired by running the same heating–cooling–heating consecutive cycles of the cells, both enclosing an empty pan–lid capsule. Therefore, in the second heating cycle, sample-mass-normalized, baseline-subtracted heat flow (W g^−1^) was plotted against temperature (°C) to constitute the DSC thermograms of the samples for analysis. In each thermogram, the inflection point (°C) of any glass-transition step(s) was identified as a *T*_g_.

### 2.14. Vicat Softening Temperature Testing

The *VST*s of the plasticated SCA, as-prepared SCA–Zn, and PS/SCA–Zn blends (including the plasticated PS) were measured under a load of 10 N in accordance with the ISO 306 testing standard (method A50) using a heat deflection and Vicat softening temperatures tester (Shenzhen City Aode Saichuang Technology Co., Ltd., China, Auto-RBWK), during their heating in a methyl-silicone oil bath from 25 to 200 °C at a rate of 50 °C h^−1^. The well-dried sample powders were compression-molded at 10 MPa at 260 °C for the SCA–Zn and 220 °C for the others, into a large, 4 mm-thick sheet that was then cut into standard rectangular (10 × 10 × 4 mm) specimens for the testing. To ensure statistical significance, the arithmetic means of three parallel *VST* results were taken as the data for analysis.

### 2.15. Mechanical Properties Testing

The mechanical properties of the PS/SCA–Zn blends were researched against those of the plasticated PS. The tensile properties were measured on a universal testing machine (MTS (Shenzhen, China), CMT-4202) in the light of the ISO 527 testing standard, using approximately 2 mm-thick dumbbell-shaped specimens with a gauge length of 20 mm and a crosshead speed of 2 mm min^−1^. In each of the stress–strain curves, the fracture strength was defined as the tensile strength, since none of them exhibited a yield behavior. The notched impact strength was tested using a Charpy impact tester (Chengde City Testing Machinery Co., Ltd., China, XJJ-50) according to the ISO 179-1 testing standard (specimen type 1). An injection molding machine (Hangzhou City Dayu Machinery Co., Ltd., China, TY-200) was used to mold the standard tensile and impact specimens from the well-dried sample powders, with three-sectional barrel and mold temperatures, respectively, of 230, 235, 240 °C, and RT and injection, holding, and cooling times of 3, 20, and 30 s, respectively. To ensure statistical significance, the arithmetic means of five parallel measurements were regarded as the data for discussion in Young’s modulus, tensile strength, elongation at break, and impact strength.

## 3. Results

### 3.1. Evidence of Success in Synthesizing Low (Half Critical) Molecular Weight High Ion Content Zn-salt SCA Ionomer

It is seen from Table 1 that the weight-average molecular weight (${\bar{M}}_{w}$) and polydispersity index (${\bar{M}}_{w}/{\bar{M}}_{n}$) of the product resin of the suspension free-radical copolymerization, presumably an SCA, were 21,000 g mol^−1^ and 1.8 respectively, which contrast sharply with the much higher ${\bar{M}}_{w}$ (180,000 g mol^−1^) and larger ${\bar{M}}_{w}/{\bar{M}}_{n}$ (3.2) of the commercial PS (GPPS 666D). Thus, the ${\bar{M}}_{w}$ of the potential SCA was tailored to approximately half of the critical ${\bar{M}}_{w}$, i.e., 50,000 g mol^−1^ [29], of PS with respect to its ${\bar{M}}_{w}$–*T*_g_ relationship. Such a low (i.e., half critical) ${\bar{M}}_{w}$ might maximally improve the processability (i.e., melt fluidity) of the potential SCA, despite the possible CA introduction of interchain hydrogen bond cross-links, while essentially maintaining its highest possible *T*_g_. Usually, this is because the ultimate *T*_g_ (i.e., $T_{g}^{\infty }$) at ca. the critical ${\bar{M}}_{w}$ of polymers decreases little with a reduction in their ${\bar{M}}_{w}$ by half.

The CA contents, *c*_as_ and *c*_CA_, of the presumable as-synthesized and purified SCA resins, respectively, were evaluated from Equation (1) by the acid–base titration to be 13.6 and 10.8 mol % (Table 2). Subject to the reactivity ratios of styrene and CA monomers, the product resin might primarily comprise one of three possible species, PS/poly(cinnamic acid) (PCA) blend, PS, and SCA. From the resin’s purified titration sample by reprecipitation from xylene into ethanol (cf. Section 2.4), free (i.e., unpolymerized) CA and/or PCA molecules, if any, effectively were eliminated by the dissolving ethanol. Therefore, if it had been a PS/PCA blend or PS, the resin would have presented a near zero *c*_CA_ in its purified sample (basically a PS), which obviously was not the case. In other words, upon its purification (i.e., removal of any free CA from it), the resin actually showed a significant *c*_CA_ of 10.8 mol % (Table 2(b)) considering the CA-comonomer feed ratio of 20 wt % (approximately 15.0 mol %), which preliminarily indicates success in the copolymerization trial of CA into PS to form an SCA. It is also noteworthy that the potential as-synthesized SCA had a *c*_as_ of 13.6 mol % (Table 2(a)), suggesting that the residual, free CA molecules present in it accounted for approximately 2.8 mol %.

**Figure 1:**
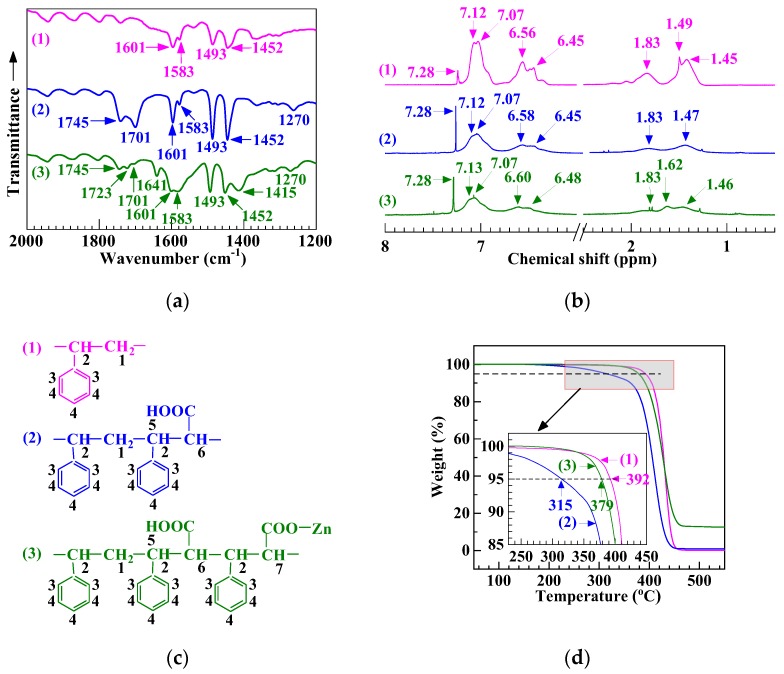
(**a**) Fourier transform infrared absorption spectra, (**b**) ^1^H solution-state nuclear magnetic resonance (NMR) spectra, (**c**) schematic chemical structures with H atoms numbered, and (**d**) thermogravimetric traces at a rate of 20 °C min^−1^ for (1) a commercial polystyrene (PS), (2) supposedly a low (i.e., half critical) molecular weight poly(styrene–*ran*–cinnamic acid) (SCA), containing 10.8 mol % of cinnamic acid, synthesized by suspension free-radical copolymerization, and (3) presumably a low molecular weight, high ion content SCA Zn salt (SCA–Zn) prepared by melt neutralization of the potential SCA with excess ZnO. All of the samples in (**a**), (**b**), and (**d**) are purified by reprecipitation from xylene into ethanol.

According to Section 2.5, supposedly an SCA–Zn was then prepared by melt neutralization of the presumable as-synthesized SCA with an excess (2.5 times the stoichiometric amount) of ZnO. Shown in Figure 1a are the FTIR absorption spectra of the potential (2) SCA and (3) SCA–Zn against (1) the PS (GPPS 666D). Note that, for PS-based chain structural verification purposes, all of the three FTIR resin samples used were purified versions (cf. Section 2.6) to remove therefrom impurities, CA, PCA, their Zn salts, etc. species. In Trace 1 of the PS, the bands at 1601, 1583, 1493, and 1452 cm^−1^ were attributed to the stretches of the phenyl C–C bonds [30,31]. Trace 2 for the potential SCA exhibited, besides all of the above bands, new peaks at 1745, 1701, and 1270 cm^−1^, of which the former two arose from the stretches of free and (cyclic) dimeric carboxyl C = O bonds, respectively [7], and the latter arose from the stretch of carboxyl C–O bonds [32,33]. This reveals success in synthesizing SCA resin by the suspension free-radical copolymerization of styrene and CA. Further, Trace 3 of the potential SCA–Zn showed, apart from all of the Trace 2 features, new bands at 1723, 1641, and 1415 cm^−1^, of which the former was ascribed to the stretch of noncyclic dimeric carboxyl C = O bonds [7], and the latter two, respectively, were ascribed to the antisymmetric and symmetric stretches of the C–O bonds of carboxylate groups (i.e., –COO^−^ anions coordinating metal cations) [34,35]. This discloses that the potential SCA successfully was melt neutralized partly to produce an SCA–Zn. As the 1641 and 1415 cm^−1^ characteristic absorptions were relatively strong, the degree of neutralization (and thus the effective ion content), although difficult to quantify due to the presence of residual, unreacted ZnO in the purified sample, appeared to be rather high in the potential SCA–Zn as a result of the excess ZnO feed.

Likewise, the same PS, potential SCA, and SCA–Zn samples purified were used for ^1^H NMR spectroscopy (cf. Section 2.7) and TGA (cf. Section 2.8) to further verify their chain structures. Figure 1b shows the ^1^H NMR spectra of the potential (2) SCA and (3) SCA–Zn relative to (1) the PS. Numbered in Figure 1c are the supposed H atoms of the three resins, to which their ^1^H peak chemical shift (*δ*) values in Figure 1b were assigned as given in Table 3. Note that in Figure 1b(1)–(3), there were 1.49 and/or 7.28 ppm peak, respectively, of the residual H_2_O and/or CHCl_3_ in the chloroform-*d* solvent [36]. In Figure 1c(1) versus 1b(1) of the PS, the peaks of the H atoms of its CH_2_ (no. 1), aliphatic CH (no. 2), phenyl ortho-(no. 3), meta- and para- (no. 4) CH, respectively, appeared at *δ* values of 1.45, 1.83, 6.45 and 6.56, and 7.07 and 7.12 ppm [37]. Compared with the PS, the potential SCA in Figure 1c(2) versus 1b(2) did not exhibit any new peak(s) for its carboxyl hydrogens (no. 5) as well as their neighboring α-H atoms (no. 6), which presumably was due to the high activity of the carboxyl groups with respect to H atoms [38]. However, contrasted with the potential SCA, the presumable SCA–Zn demonstrated, for H-atoms no. 7, an additional new peak at 1.62 ppm (cf. Figure 1c(3) versus 1b(3)). This had to be owing to the Zn electrophilic substitution of part of the carboxyls at their H atoms during the neutralization, which deactivated some of the α-H atoms (no. 6) into those (no. 7) that displayed their peak at 1.62 ppm [39]. The full reconciliation of Figure 1b,c in Table 3, discussed above, verifies that the SCA–Zn synthetic trial was successful. Since the 1.62 ppm peak was pronounced enough in Figure 1b(3), the (effective) ion content, dictated by the degree of neutralization, of the potential SCA–Zn should be rather high, especially considering the 10.8 mol % of CA partly neutralized by the excess ZnO (2.5 times the stoichiometric amount).

Figure 1d gives the TGA thermograms of the three resins, indicating the thermal decomposition behaviors of the potential (2) SCA and (3) SCA–Zn as opposed to (1) the PS. It is seen that compared with the PS *T*_d_ of 392 °C, the potential SCA *T*_d_ sharply was reduced to 315 °C, which was likely due to its much lower (i.e., half critical) ${\bar{M}}_{w}$ (cf. Paragraph 1, Section 3.1) as well as susceptibility to decarboxylation with CO_2_ evolution [40]. Nevertheless, upon the melt neutralization, the *T*_d_ of the potential SCA–Zn rebounded greatly to 379 °C approximating to that of the PS, which was probably thanks to a significant decrease in the vulnerable carboxyls fraction and hence dense ionic cross-linking resembling an ${\bar{M}}_{w}$ increase effect. The above TGA discussions further prove that a high ion content SCA–Zn, having a half critical ${\bar{M}}_{w}$ inherited from its SCA precursor, successfully was synthesized.

Finally, the same probable SCA and SCA–Zn samples purified were subjected to intrinsic viscosity ([*η*]) measurement to confirm the success in the SCA–Zn synthesis; the [*η*] of the PS was not tested for the comparative studies due to its much higher (i.e., commercial) ${\bar{M}}_{w}$. It was found from Table 4 that upon the melt neutralization, the [*η*] of the likely SCA–Zn was reduced considerably until 0.162 dL g^−1^ from that (0.197 dL g^−1^) of the likely SCA. Considering the same degree of polymerization (i.e., chain length) of the two resins, this had to be ascribed to a remarkable occurrence of intramolecular ionic cross-links through the significant introduction of Zn-carboxylate ionic groups, which shrank a single coiled SCA–Zn macromolecular chain in radial size to a large extent. All of the above contexts, including the GPC, titration, FTIR, NMR, TGA, and [*η*] results, corroborate conclusively that a low (i.e., half critical) ${\bar{M}}_{w}$, high ion content SCA–Zn ionomer, indeed, was synthesized successfully by the styrene–CA suspension free-radical copolymerization followed by the excess ZnO melt neutralization.

### 3.2. Minimization of a Processability Mismatch between PS and the SCA Zn Salt during their Melt Blending

**Figure 2:**
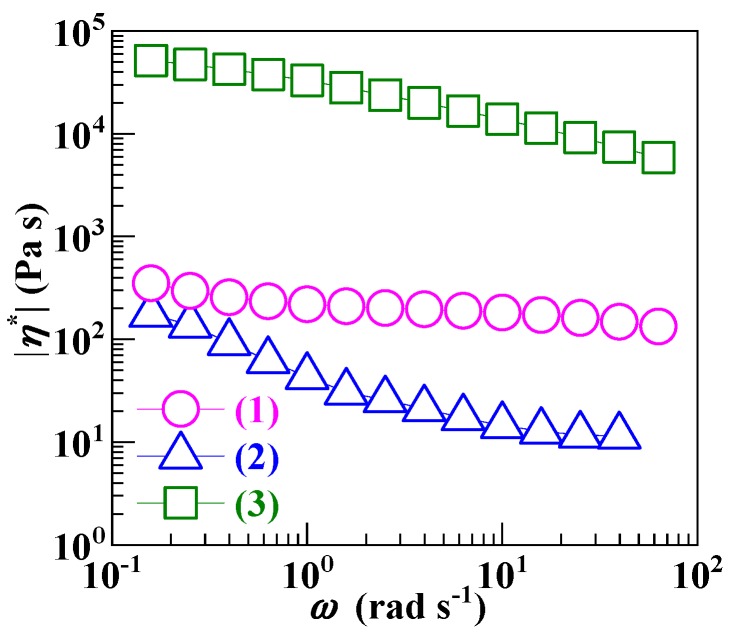
Complex viscosity magnitudes (|η^*^|’s) as functions of angular frequency (*ω*), i.e., log|η^*^| versus log*ω* curves, in the oscillatory shear mode at 220 °C and a strain amplitude of 0.5% for (1) a commercial polystyrene, (2) a low (i.e., half critical) molecular weight poly(styrene–*ran*–cinnamic acid) (SCA), bearing 10.8 mol % of cinnamic acid, synthesized by suspension free-radical copolymerization, and (3) a low molecular weight, high ion content SCA Zn salt prepared by melt neutralization of the SCA with excess ZnO.

Figure 2 illustrates the complex viscosity magnitudes (|η^*^|’s) at 220 °C, during angular frequency (*ω*) sweep tests, of the (2) SCA and (3) SCA–Zn against those of (1) the PS. In the light of the Cox–Merz rule [41] shown below by Equation (4),

(4) $$|\eta^{*}|{|}_{\omega =\dot{\gamma }}=\eta$$

the obtained |η^*^|–*ω* traces in the oscillatory shear mode are reduced to flow curves of shear viscosity (*η*) versus shear (strain) rate ($\dot{\gamma }$) in the steady-state shear mode. It is observed that across the range of (10^−1^–10^2^ s^−1^) $\dot{\gamma }$ values investigated, the *η* values (Trace 2) of the SCA all fell below those (Trace 1) of the PS, which apparently resulted from competition between two effects. One effect, positive, was the sharp ${\bar{M}}_{w}$ lowering of the SCA until the half critical ${\bar{M}}_{w}$ of PS (Table 1), which dramatically reduced its melt *η* from that of the (commercial) PS. While the other effect, negative, should be the dense hydrogen bond cross-linking along with the rigid, polar copolymerization of the SCA by 10.8 mol % of CA (Table 2), which significantly increased its melt *η*. Obviously, the positive effect predominated over the negative to give a very low *η* precursor, the half critical ${\bar{M}}_{w}$ SCA, to its high ion content ionomer. In addition, note that the SCA (Trace 2) presented a more distinct shear thinning behavior than the PS (Trace 1), which was presumably a consequence of progressive rupture of its hydrogen bond cross-links with increasing $\dot{\gamma }$.

Upon the melt neutralization, the *η* values (Trace 3) of the high ion content SCA–Zn greatly were enhanced by ca. 2.5–3.0 orders of magnitude in contrast to those (Trace 2) of the SCA. This essentially was due to the introduction, into the SCA–Zn, of dense, ionic Zn-carboxylate triplet and aggregate cross-links, which are much higher in bond strength than the hydrogen bond cross-links in the SCA. As a result, the SCA–Zn’s *η* values (Trace 3) in turn lay well (ca. 1.5–2.0 orders of magnitude) above those (Trace 1) of the PS at all of the $\dot{\gamma }$ values studied (Figure 2). Nevertheless, the *η* differences between the SCA–Zn and PS already were strikingly smaller than if the SCA precursor had been of commercial ${\bar{M}}_{w}$, when its *η*–$\dot{\gamma }$ trace would have risen above that (Trace 1) of the PS and become near parallel to that (Trace 2) of the half critical ${\bar{M}}_{w}$ SCA with possible *η* differences of at least 2.0 orders of magnitude. In other words, tailoring the SCA to its half critical ${\bar{M}}_{w}$ and thereby retaining its *T*_g_ close to $T_{g}^{\infty }$ minimized the *η* differences (i.e., processability mismatches) between the SCA–Zn (Trace 3) and PS (Trace 1) while, basically, it did not compromise the former’s potential to improve the latter’s heat resistance during their melt blending. It is particularly worth noting that the SCA–Zn (Trace 3) displayed milder shear thinning than the SCA (Trace 2), which was likely due to its introduction of stronger ionic cross-links that, unlike hydrogen bond cross-links, were not prone to irreversible disruption at all with increasing $\dot{\gamma }$. Regardless of this, the *η* difference at a terminal $\dot{\gamma }$ of 10^2^ s^−1^, approximate to the average $\dot{\gamma }$ during the plasticorder melt blending (Section 2.10), was merely approximately 1.5 orders of magnitude between the half critical ${\bar{M}}_{w}$, high ion content SCA–Zn (Trace 3) and PS (Trace 1), which made the melt blending of PS and SCA–Zn possible.

**Figure 3:**
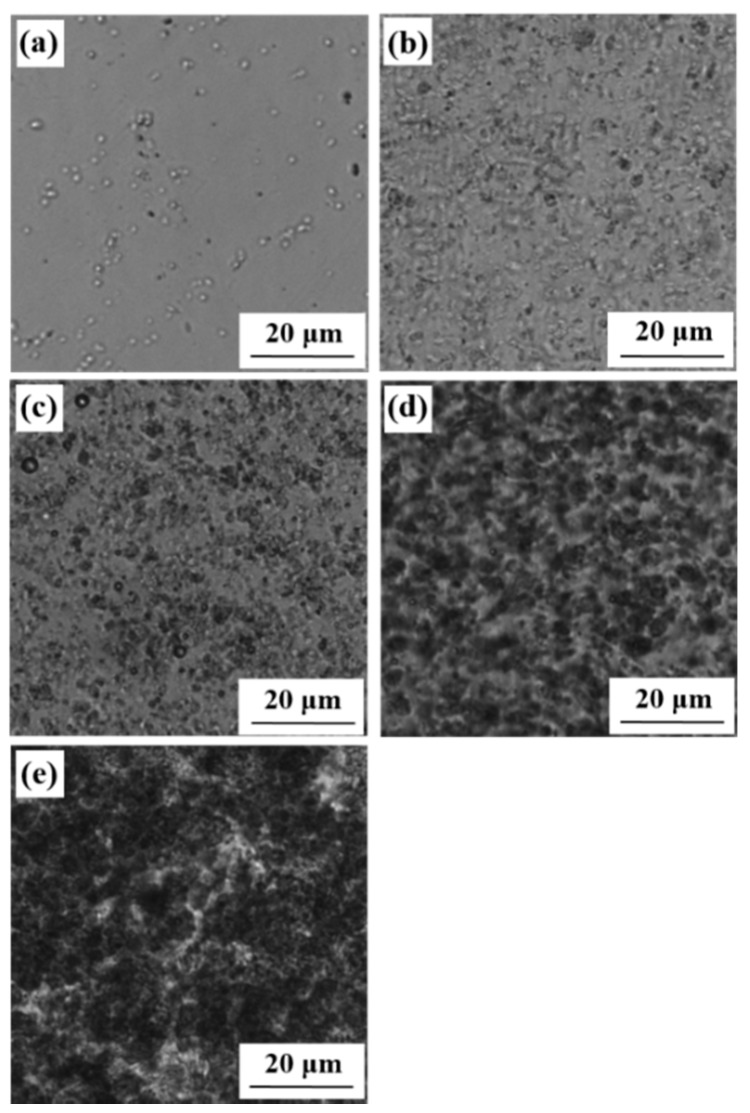
Optical micrographs, taken at 220 °C, showing the phase morphologies of (**a**) a commercial polystyrene (PS) and (**b**)–(**e**) blends that are prepared by melt blending of the PS with increasing mass fractions (5–40 wt %) of a low (i.e., half critical) molecular weight, high ion content poly(styrene–*ran*–cinnamic acid) Zn salt (SCA–Zn): (**b**) PS/SCA–Zn (95/5); (**c**) PS/SCA–Zn (90/10); (**d**) PS/SCA–Zn (80/20); (**e**) PS/SCA–Zn (60/40).

In the above context, the PS and SCA–Zn were then melt-blended, at 220 °C and an 80 rpm rotor speed, at five decreasing mass ratios of 100/0 (i.e., plasticated PS), 95/5, 90/10, 80/20, and 60/40 (Section 2.10). To examine the effect of the processability mismatch minimization, the morphologies of the PS/SCA–Zn blends were observed at 220 °C by optical microscopy, as illustrated in Figure 3, where the dark domains represented the SCA–Zn discrete phase while the bright background represented the PS matrix in each of the images. Apparently, all of the blends, except for the neat PS (Figure 3a), had a fine two-phase morphology of SCA–Zn particles delicately dispersed in a PS matrix with very blurred phase boundaries. This indicates that the PS–(SCA–Zn) processability mismatch presumably was minimized to result in acceptable interfacial compatibility and hence interfacial adhesion of the blends. Scrutinization of Figure 3b–e further reveals that, dramatically, the particle size of the SCA–Zn became steadily larger with a monotonic increase in its content (5–40 wt %), suggesting that the interfacial compatibility might deteriorate significantly with increasing the SCA–Zn content of the blends.

**Figure 4:**
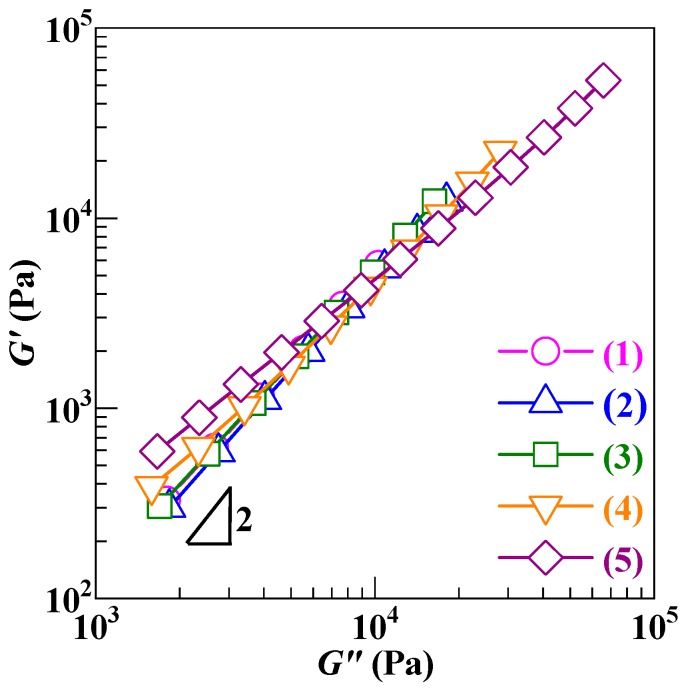
Han (i.e., log*G*′ versus log*G*″) plots, which are obtained from frequency sweep tests at 220 °C and a strain amplitude of 0.5% in the oscillatory shear mode, for (1) a commercial polystyrene (PS) and (2)–(5) melt blends of the PS and increasing mass fractions (5–40 wt %) of a low (i.e., half critical) molecular weight, high ion content poly(styrene–*ran*–cinnamic acid) Zn salt (SCA–Zn): (2) PS/SCA–Zn (95/5); (3) PS/SCA–Zn (90/10); (4) PS/SCA–Zn (80/20); (5) PS/SCA–Zn (60/40).

More quantitatively, the effect of the processability mismatch minimization was probed by a rheological oscillatory shear approach. Shown in Figure 4 are the Han (i.e., log*G*′ versus log*G*″) plots (Traces 2–5) of the PS/SCA–Zn blends against that (Trace 1) of the (neat) PS at 220 °C. It is observed that, similar to the PS (Trace 1), the Han plots of the 95/5 (Trace 2) and 90/10 (Trace 3) blends both demonstrated a slope of approximately 2 in the lower *ω* (i.e., lower left) terminal region, which was indicative of their almost homogeneous (i.e., single) phase morphology [42]. This discloses that instead of their apparent two-phase morphologies shown in Figure 3b,c respectively, both of the 95/5 and 90/10 blends actually were near miscible at 220 °C, which constitutes strong evidence that the PS–(SCA–Zn) processability mismatch indeed was minimized using the half critical ${\bar{M}}_{w}$ SCA precursor. However, as the SCA–Zn content was raised further beyond 10 wt % more and more, the resulting 80/20 (Trace 4) and 60/40 (Trace 5) blends had Han-plot terminal slopes increasingly divergent from (i.e., smaller than) 2, revealing their gradually heterogeneous (i.e., two-phased) morphologies [43]. On the whole, with a monotonous rise in their SCA–Zn content, the miscibility of the blends progressively was reduced, which corresponds to the steady decrease in their interfacial compatibility apparently observed from Figure 3.

### 3.3. An Effective Improvement in the Heat Resistance of PS upon its Blending Modification with a Significant Fraction of the SCA Zn Salt

**Figure 5:**
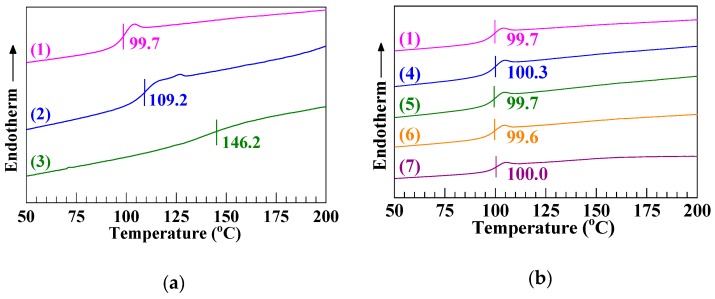
(**a**) Differential scanning calorimetry thermograms in the second heating cycle at a rate of 20 °C min^−1^ for (1) a commercial polystyrene (PS), (2) a low (i.e., half critical) molecular weight poly(styrene–*ran*–cinnamic acid) (SCA), bearing 10.8 mol % of cinnamic acid, synthesized by suspension free-radical copolymerization, and (3) a low molecular weight, high ion content SCA Zn salt (SCA–Zn) prepared by melt neutralization of the SCA with excess ZnO. (**b**) Those for (1) the PS and (4)–(7) melt blends of the PS and increasing mass fractions (5–40 wt %) of the SCA–Zn: (4) PS/SCA–Zn (95/5); (5) PS/SCA–Zn (90/10); (6) PS/SCA–Zn (80/20); (7) PS/SCA–Zn (60/40).

Figure 5a and Table 5(a–c), respectively, give the *T*_g_ and *VST* values of the PS, SCA, and SCA–Zn. It is seen that the *T*_g_ and *VST* (109.2 and 108.2 °C), respectively, of the SCA were approximately 10 °C higher than those (99.7 and 97.7 °C) of the PS. This primarily was from the chain-segmental hydrogen bond cross-linking as well as rigid, polar copolymerization of the SCA by 10.8 mol % of CA, since the SCA’s heat resistance should little be reduced, from that of the (commercial) PS, by its half critical ${\bar{M}}_{w}$. More dramatically, the *T*_g_ and *VST* of the SCA–Zn were enhanced to 146.2 and 153.1 °C, respectively, from those of the SCA by 37 and 45 °C, which obviously was due to its significant ionization to form dense, ionic (i.e., Zn-carboxylate) chain-segmental cross-links much stronger in strength than hydrogen bond cross-links. Interestingly, upon the 10 N loading and slower (50 °C h^−1^) heating (cf. Section 2.14), while the PS and SCA displayed their *VST*s (97.7 and 108.2 °C), respectively, which were slightly lower than the *T*_g_ values (99.7 and 109.2 °C), the SCA–Zn showed an opposite trend; its *VST* (153.1 °C) was considerably higher than *T*_g_ (146.2 °C). This infers that the high ion content SCA–Zn likely had, according to the multiplet-cluster model [44], a two-phase morphology of matrix phase (*T*_g_ = 146.2 °C) and stiffer cluster phase whose higher *T*_g_ was difficult to discern by DSC. As such, the *VST*, a morphological behavior of the SCA–Zn should be intermediate between the *T*_g_ values of its matrix and cluster phases, i.e., higher than its matrix *T*_g_.

Figure 5b and Table 5(a,d–g), respectively, show the *T*_g_ and *VST* values of the PS/SCA–Zn melt blends ranging from 100/0 (i.e., the PS) to 60/40. It is found from Figure 5b that the four blends (Traces 4–7) all exhibited almost the same *T*_g_ as the PS (Trace 1), approximately 100 °C, which arose typically from the phase behavior nature of glass transition. In the near miscible (cf. Figure 4) 95/5 and 90/10 blends, the SCA–Zn chain-segmental ionic cross-links essentially were dissolved (i.e., destructed) by the major PS chains, making the minor SCA–Zn rigidification of the PS negligible (Traces 4 and 5, Figure 5b); even though the two blends were phase-separated near 100 °C (their near miscibility was observed from Figure 4 merely at 220 °C), a single *T*_g_ probably occurred for the PS matrix phase with the matrix *T*_g_ of the minor SCA–Zn phase indiscernible. Likewise, for the apparently two-phased (cf. Figure 4) 80/20 and 60/40 blends, there appeared only one *T*_g_ of the PS matrix phase despite the larger SCA–Zn fractions (Traces 6 and 7, Figure 5b), which was presumably due to the significantly weaker glass-transition step (Trace 3, Figure 5a) of the SCA–Zn’s matrix phase than that (Trace 1, Figure 5a) of the PS. Altogether, the two-phased PS/SCA–Zn blends (i.e., Traces 4–7 or 6 and 7, Figure 5b) invariably demonstrated a PS-matrix *T*_g_ little shifting from that of the neat PS (Trace 1, Figure 5b) toward a higher temperature, suggesting that their claimed acceptable interfacial compatibility at 220 °C (Figure 3) actually was not good enough at approximately 100 °C to give any convergence of the two components’ *T*_g_ values (99.7 and 146.2 °C).

**Figure 6:**
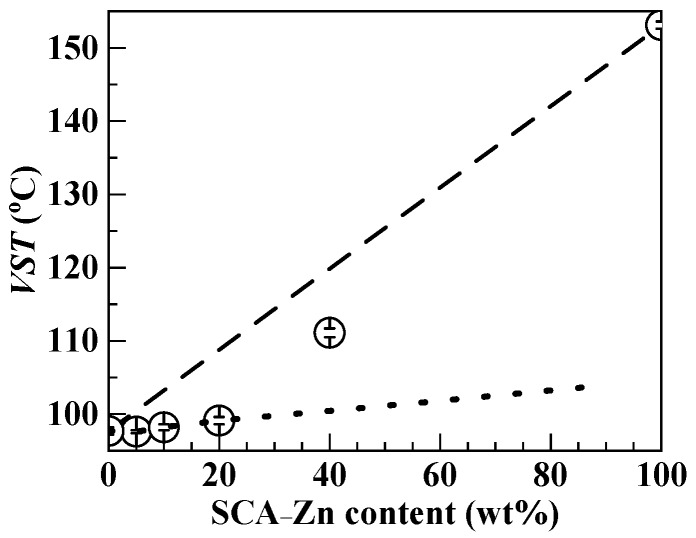
Dependence of Vicat softening temperature (*VST*) upon the SCA–Zn content of the PS/SCA–Zn melt blends (including the neat PS), which is plotted using the data from Table 5(a,d–g).

Nevertheless, similar to that (Table 5(c)) of the SCA–Zn, the *VST* data (Table 5(a,d–g)) of the blends had to be interpreted in the context of the morphological behavior character of softening. That is, contingent upon their miscibility and composition, the single *VST* of the blends should be dictated by a weighted combination of the *VST* values, 97.7 and 153.1 °C respectively, of their components, PS and SCA–Zn. To elucidate this straightforwardly, the heat resistance (i.e., *VST*) was plotted against the SCA–Zn content of the blends as shown in Figure 6, using the Table 5(a,c–g) data. It is apparent that, with increasing the SCA–Zn content up to 20 wt %, the *VST* was raised satisfactorily linearly, as well as quite slightly given the much steeper slope (i.e., dashed line) between the PS and SCA–Zn *VST*s (97.7 and 153.1 °C respectively). This likely infers that the PS/SCA–Zn (95/5, 90/10, and 80/20) blends all were more or less near miscible at approximately 100 °C, since miscible blends frequently feature a linear composition–property empirical relationship [45] and that only when the blends basically were miscible could the strong, dense ionic cross-links of the minor SCA–Zn thoroughly be dissolved (i.e., destructed) by the major PS to pose the markedly milder linearity slope (i.e., dotted line) than the dashed line. In other words, the SCA–Zn rigidification of the PS was rather weak in the miscible blends where there primarily remained a slightly stiffer CA and CA–Zn copolymerization effect due to the C–C bond internal rotation limitation by the steric hindrance of carboxyl and Zn-carboxylate groups.

As the SCA–Zn content further was enhanced to 40 wt %, the situation of the blend changed in that its *VST* conspicuously diverged, positively (i.e., upwards), from the dotted-line linearity until approximately 111.1 °C (Figure 6 and Table 5(g)), which was approximately 13.5 °C higher than that (97.7 °C) of the PS. This indicates that the PS/SCA–Zn (60/40) blend distinctly was phase-separated at approximately 110 °C into a two-phase morphology, probably, of rigid (*VST* = 153.1 °C) SCA–Zn particles dispersed in a far more flexible (*VST* = 97.7 °C) PS matrix. Meanwhile, the 111.1 °C *VST* data point fell below the dashed-line linearity, revealing that the blend’s interfacial compatibility was unsatisfactory at approximately 110 °C [46], which agrees with the DSC observation of its not good enough interfacial compatibility at approximately 100 °C (Trace 7, Figure 5b). Generally, the *VST*s data (Figure 6) demonstrated stepwise reduced miscibility with raising the SCA–Zn content of the blends, which, for the most part, is consistent with the microscopic and rheological results observed from Figure 3 and Figure 4, respectively. In summary, across the SCA–Zn contents (0–40 wt %) studied, not until it was as significant at up to 40 wt % did the PS/SCA–Zn blend evolve a typical two-phase morphology despite insufficient interphase compatibility, in which the strong, dense ionic Zn-carboxylate cross-links of the dispersed SCA–Zn phase contributed primarily to the effective improvement in the blend’s heat resistance (i.e., *VST* increase by approximately 13.5 °C).

### 3.4. Deteriorations in the Mechanical Strengths of PS upon its Incorporation of the SCA Zn Salt

**Figure 7:**
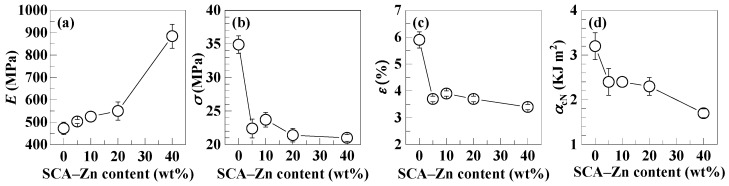
Effects of SCA–Zn content on the (**a**) Young’s modulus (*E*), (**b**) tensile strength (*σ*), (**c**) elongation at break (*ε*), and (**d**) Charpy notched impact strength (*α*_cN_) of the PS/SCA–Zn melt blends (including the neat PS), which are plotted using the data given in Table 6.

While the half critical ${\bar{M}}_{w}$, densely ionically cross-linked SCA–Zn, at a 40 wt % fraction, minimized its processability mismatch with the PS and significantly enhanced the heat resistance of the phase-separated PS/SCA–Zn blend, it was imperative to examine the mechanical and rheological properties of the blend against the PS from a polymer engineering viewpoint. Table 6 lists the mechanical properties, i.e., Young’s moduli (*E*), tensile strengths (*σ*), elongations at break (*ε*), and Charpy notched impact strengths (*α*_cN_), of the PS/SCA–Zn blends having the increasing SCA–Zn contents (0–40 wt %), from which the composition–property relationships were plotted as illustrated in Figure 7. It is seen from Figure 7a that the *E* heightened first linearly and moderately with increasing the SCA–Zn content up to 20 wt %, and then it heightened nonlinearly and sharply as the SCA–Zn content was raised further to 40 wt %. This behavior, analogous to that of the *VST* change with the SCA–Zn content shown in Figure 6, seems to verify that for similar reasons, the blends were near miscible at the SCA–Zn contents of ≤ 20 wt %, whereas they were distinctly phase-separated with insufficient interfacial compatibility at 40 wt % at RT. However, the *σ*, *ε*, and *α*_cN_ values all had a general tendency to decrease steadily with a monotonic rise in the SCA–Zn content of the blends (Figure 7b–d, respectively). This possibly was owing to the mechanical weakness and brittleness of the dissolved low-${\bar{M}}_{w}$ SCA–Zn without ionic cross-links or polar chain-segmental interactions in the miscible blends (95/5, 90/10, and 80/20), as well as to primarily the unsatisfactory interfacial compatibility that caused insufficient interphase adhesion in the two-phased blend (60/40). Note that for the miscible blends without interfaces, while the *E* was enhanced primarily from the rigid CA and CA–Zn copolymerization on a mer scale, the *σ*, *ε*, and *α*_cN_ were decreased due essentially to the SCA–Zn’s low ${\bar{M}}_{w}$ (i.e., plasticization and embrittlement) on a wholly macromolecular scale. Further, for the 60/40 phase-separated blend with the strong ionic cross-links, unlike the *E* insusceptibility to interfacial softening upon small strain and stress, all of the *σ*, *ε*, and *α*_cN_ values were quite vulnerable to a deterioration from the low-strength interphase upon much larger strain and stress. Consequently, compared with the *E*, *σ*, *ε*, and *α*_cN_ (472.6 MPa, 34.9 MPa, 5.9%, and 3.2 kJ m^−2^ respectively) of the PS, the *E* was enhanced until 884.6 MPa by 87.2%, while the *σ*, *ε*, and *α*_cN_ were decreased to 21.0 MPa, 3.4%, and 1.7 kJ m^−2^, respectively, by 39.8%, 42.4%, and 46.9% for the PS/SCA–Zn (60/40) blend (cf. Table 6). These results show that the SCA–Zn 40 wt % incorporation appreciably improved the heat resistance of the PS/SCA–Zn blend essentially at the expense of its mechanical (tensile and impact) properties due to the two phases insufficiently adhering to each other.

### 3.5. A Controlled Reduction in the Processability with Increasing the SCA Zn Salt Content of the PS/SCA Zn Salt Blends

**Figure 8:**
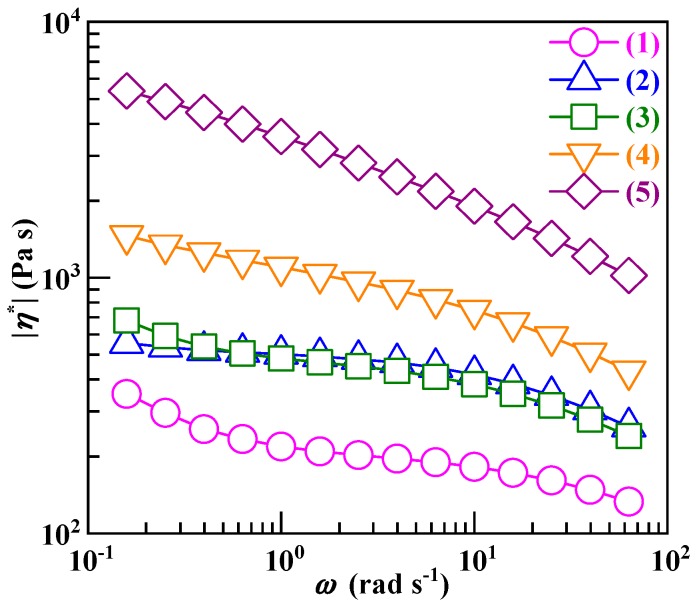
Complex viscosity magnitudes (|η^*^|’s) as functions of angular frequency (*ω*), i.e., log|η^*^| versus log*ω* curves, in the oscillatory shear mode at 220 °C and a strain amplitude of 0.5% for (1) a commercial polystyrene (PS) and (2)–(5) melt blends of the PS and increasing mass fractions (5–40 wt %) of a low (i.e., half critical) molecular weight, high ion content poly(styrene–*ran*–cinnamic acid) Zn salt (SCA–Zn): (2) PS/SCA–Zn (95/5); (3) PS/SCA–Zn (90/10); (4) PS/SCA–Zn (80/20); (5) PS/SCA–Zn (60/40).

It generally is observed from Figure 8 that, according to the Cox–Merz rule (Equation (4)), the *η* values at 220 °C became increasingly higher with a steady rise in the SCA–Zn fraction of the PS/SCA–Zn blends over the $\dot{\gamma }$ values investigated. The *η* values (Trace 2) of the 95/5 blend were all comparable to those (Trace 3) of the 90/10 blend, which could be due to their both near miscible morphologies (cf. Figure 4, Figure 6, and Figure 7a) where the small fraction of the SCA–Zn was dissolved in the PS to completely disengage its strong, dense ionic cross-links. All of the five blends (including the PS) exhibited a shear thinning behavior regardless of their homogeneous (Traces 1–3) or two-phased (Traces 4 and 5) morphologies. At the terminal $\dot{\gamma }$ of approximately 10^2^ s^−1^, the *η* (Trace 5) of the 60/40 blend was just approximately 1 order of magnitude higher than that (Trace 1) of the PS, which was as low as approximately 10^2^ Pa s, indicating its superior melt fluidity; this accords with the processability mismatch minimization (i.e., *η* difference of approximately 1.5 orders of magnitude) between the SCA–Zn and PS at the same $\dot{\gamma }$ (Figure 2). In other words, the high ion content (i.e., dense ionic cross-links) of the SCA–Zn incorporated at 40 wt % significantly improved the heat resistance of the PS/SCA–Zn blend (Table 5(a) versus (g) and Figure 6) without markedly sacrificing its excellent processability primarily as a result of the SCA–Zn’s half critical ${\bar{M}}_{w}$.

## 4. Conclusions

A low (i.e., half critical) ${\bar{M}}_{w}$ (approximately 21,000 g mol^−1^), high ion content Zn-salt carboxylated PS ionomer, SCA–Zn, has been successfully synthesized by styrene–CA (10.8 mol %) suspension free-radical copolymerization followed by excess (2.5 times the stoichiometric amount of) ZnO melt neutralization, as evidenced by GPC, acid–base titration, FTIR and NMR spectroscopic, TGA, and intrinsic viscosity measurement results. As observed by oscillatory shear rheometry and optical microscopy both at 220 °C, the *η* of the densely, ionically cross-linked SCA–Zn merely is approximately 1.5 orders of magnitude higher than commercial (${\bar{M}}_{w}$ approximately 180,000 g mol^−1^) PS at a representative $\dot{\gamma }$ of 10^2^ s^−1^, melt blends of the PS and SCA–Zn (5–40 wt %) all exhibit an apparently fine, two-phased morphology with blurred phase boundaries indicating acceptable interfacial compatibility, and the Han plots of the PS/SCA–Zn (95/5 and 90/10) blends reveal their actually near miscible (i.e., single-phase) morphologies. These findings prove that the PS–(SCA–Zn) processability mismatch, if any, presumably is minimized by means of the SCA–Zn’s half critical ${\bar{M}}_{w}$ value, despite its strong ionic cross-links. Since the ultimate *T*_g_ at ca. the critical ${\bar{M}}_{w}$ of polymers reduces little with a decrease in their ${\bar{M}}_{w}$ by half, the high ion content SCA–Zn’s *VST* essentially is maximized to 153.1 °C from that (97.7 °C) of the PS in terms of the effect of its half critical ${\bar{M}}_{w}$. For the PS/SCA–Zn (0–20 wt %) blends, the *VST* is then found to rise linearly and slightly with the SCA–Zn content (from 97.7 °C of the PS to 99.1 °C of the 80/20 blend). This suggests that they are all near miscible in the proximity of 100 °C when the SCA–Zn’s strong ionic cross-links primarily are dissolved (i.e., destructed) to leave much weaker PS rigidification by the slightly stiffer CA and CA–Zn copolymerization. As the SCA–Zn fraction further is increased to 40 wt %, the blend’s *VST* remarkably diverges positively from the 0–20 wt % linearity until 111.1 °C but falls below the linearity between the PS and SCA–Zn *VST*s. This infers that the 60/40 blend is phase-separated at approximately 110 °C into a two-phase morphology where, despite insufficient interfacial compatibility, the strong, dense ionic cross-links of the SCA–Zn particles lead basically to the effective enhancement of the blend’s *VST* (from 97.7 to 111.1 °C) by approximately 13.5 °C. Therefore, over the SCA–Zn fractions (5–40 wt %) investigated, only at 40 wt % does the half critical ${\bar{M}}_{w}$, high ion content SCA–Zn minimize its processability mismatch with the PS during their melt blending while meanwhile considerably improve the heat resistance of the phase-separated PS/SCA–Zn blend. Mechanically compared with the PS, although the *E* of the PS/SCA–Zn (60/40) blend notably is heightened by 87.2% (884.6 versus 472.6 MPa) because of its insusceptibility to interfacial softening upon small strains, both the *σ* and *α*_cN_ noticeably are lowered from 34.9 MPa and 3.2 kJ m^−2^, respectively, to 21.0 MPa and 1.7 kJ m^−2^ by 39.8% and 46.9% due to their vulnerability to deterioration mostly from the unsatisfactory interfacial adhesion upon much larger strains. Rheologically against the PS, the reduction in the processability of the PS/SCA–Zn (60/40) blend is controlled at an increase in the *η* (at 220 °C and approximately 10^2^ s^−1^) by only approximately 1 order of magnitude, which is largely a consequence of the SCA–Zn’s half critical ${\bar{M}}_{w}$. This work, from a practical melt processing viewpoint, offers insight into a viable methodology of effectively improving the heat resistance of commercial PS by melt blending modification with its half critical ${\bar{M}}_{w}$, high ion content ionomer.

## Figures and Tables

**Table 1:** (a) Weight-average molecular weight (${\bar{M}}_{w}$) and polydispersity index (${\bar{M}}_{w}/{\bar{M}}_{n}$) of a product resin, supposedly poly(styrene–*ran*–cinnamic acid) (SCA), from suspension free-radical copolymerization of styrene and cinnamic acid as opposed to (b) those of a commercial polystyrene (PS) ^a^.

Resin	${\bar{M}}_{n}$ (g mol^−1^) ^b^	${\bar{M}}_{w}$ (g mol^−1^)	$M_{p}$ (g mol^−1^) ^c^	${\bar{M}}_{w}/{\bar{M}}_{n}$
(a) (potential) SCA	12,000	21,000	19,000	1.8
(b) PS	57,000	180,000	130,000	3.2

^a^ Both the potential SCA and the PS are measured by gel permeation chromatography, respectively, in their as-synthesized and as-purchased states. ^b^ Number-average molecular weight. ^c^ Peak molecular weight.

**Table 2:** (**a**) Cinnamic acid (CA) content, *c*_as_, of presumably a low (i.e., half critical) molecular weight poly(styrene–*ran*–cinnamic acid) (SCA) as synthesized by suspension free-radical copolymerization compared with (**b**) that, *c*_CA_, of its purified version by reprecipitation from xylene into ethanol.

Resin	CA Content (mol %) ^a^
(a) (potential) as-synthesized SCA	*c*_as_ = 13.6 ± 0.1
(b) (potential) purified SCA	*c*_CA_ = 10.8 ± 0.3

^a^ Measured by acid–base titration with a titrated KOH ethanol solution, with the aid of a phenolphthalein indicator solution in an ethanol/distilled water (19/1 *v/v*) mixed solvent.

**Table 3:** Assignments of the (1) PS, potential (2) SCA, and (3) SCA–Zn’s ^1^H NMR peak chemical shift (*δ*) values shown in Figure 1b(1)–(3), respectively, to their H atoms numbered in Figure 1c(1)–(3).

H atom no.	*δ* (ppm)		
	(1) PS	(2) Potential SCA	(3) Potential SCA–Zn
1	1.45	1.47	1.46
2	1.83	1.83	1.83
3	6.45, 6.56	6.45, 6.58	6.48, 6.60
4	7.07, 7.12	7.07, 7.12	7.07, 7.13
5	– ^a^	– ^b^	– ^b^
6	– ^a^	– ^b^	– ^b^
7	– ^a^	– ^a^	1.62

^a^ These H atoms do not belong to the respective resins. ^b^ Owing to the high activity of the –COOH groups with respect to H atoms, the *δ* values of their H atoms (i.e., H-atoms no. 5) and neighboring α-H atoms (i.e., H-atoms no. 6) are not subject to detection.

**Table 4:** (a) Intrinsic viscosity ([*η*]) of likely a low (i.e., half critical) molecular weight poly(styrene–*ran*–cinnamic acid) (SCA), having 10.8 mol % of cinnamic acid, synthesized by suspension free-radical copolymerization against (b) that of its Zn salt (likely an SCA–Zn), still of low molecular weight and probably of high ion content, prepared by its melt neutralization with excess ZnO.

Resin	[*η*] (dL g^−1^) ^a^
(a) (probable) SCA	0.197
(b) (probable) SCA–Zn	0.162

^a^ Measured at 25 ± 0.1 °C in a tetrahydrofuran/distilled water (9/1 *v/v*) mixed solvent by an Ubbelohde viscometer according to the ISO 1682-1 testing standard, both using purified samples by reprecipitation from xylene into ethanol.

**Table 5:** Vicat softening temperatures (*VST*s), measured under a load of 10 N and at a heating rate of 50 °C h^−1^, for (a) a commercial polystyrene (PS), (b) a low (i.e., half critical) molecular weight poly(styrene–*ran*–cinnamic acid) (SCA), bearing 10.8 mol % of cinnamic acid, synthesized by suspension free-radical copolymerization, (c) a low molecular weight, high ion content SCA Zn salt (SCA–Zn) prepared by melt neutralization of the SCA with excess ZnO, and (d)–(g) melt blends of the PS and increasing mass fractions (5–40 wt %) of the SCA–Zn: (d) PS/SCA–Zn (95/5); (e) PS/SCA–Zn (90/10); (f) PS/SCA–Zn (80/20); (g) PS/SCA–Zn (60/40).

Composition	*VST* (°C)
(a) PS	97.7 ± 0.5
(b) SCA	108.2 ± 0.4
(c) SCA–Zn	153.1 ± 0.5
(d) PS/SCA–Zn (95/5)	97.6 ± 0.2
(e) PS/SCA–Zn (90/10)	98.2 ± 0.4
(f) PS/SCA–Zn (80/20)	99.1 ± 0.5
(g) PS/SCA–Zn (60/40)	111.1 ± 0.6

**Table 6:** Young’s moduli (*E*), tensile strengths (*σ*), elongations at break (*ε*), and Charpy notched impact strengths (*α*_cN_) for (a) a commercial polystyrene (PS) and (b)–(e) melt blends of the PS and increasing mass fractions (5–40 wt %) of a low (i.e., half critical) molecular weight, high ion content poly(styrene–*ran*–cinnamic acid) Zn salt (SCA–Zn): (b) PS/SCA–Zn (95/5); (c) PS/SCA–Zn (90/10); (d) PS/SCA–Zn (80/20); (e) PS/SCA–Zn (60/40).

Composition	*E* (MPa)	*σ* (MPa)	*ε* (%)	*α*_cN_ (kJ m^−2^)
(a) PS	472.6 ± 27.8	34.9 ± 1.3	5.9 ± 0.3	3.2 ± 0.3
(b) PS/SCA–Zn (95/5)	503.0 ± 8.9	22.4 ± 1.4	3.7 ± 0.1	2.4 ± 0.3
(c) PS/SCA–Zn (90/10)	525.1 ± 22.9	23.7 ± 1.1	3.9 ± 0.1	2.4 ± 0.0
(d) PS/SCA–Zn (80/20)	550.0 ± 40.7	21.4 ± 1.0	3.7 ± 0.1	2.3 ± 0.2
(e) PS/SCA–Zn (60/40)	884.6 ± 53.5	21.0 ± 0.5	3.4 ± 0.1	1.7 ± 0.1
